# Effectiveness of betamethasone dipropionate versus methylprednisolone acetate intra-articular injection in the management of pain in primary osteoarthritis of the knee

**DOI:** 10.4314/gmj.v59i2.6

**Published:** 2025-06

**Authors:** Emmanuel Andzie-Mensah, Michael Segbefia, Henry Holdbrook-Holdbrook-Smith, Antoinette Bediako Bowan, Jonathan Dakubo, Ambrose Agbor

**Affiliations:** 1 Greater Accra Regional Hospital. Department of Surgery, Accra, Ghana; 2 Department of Trauma and Orthopaedics, University of Ghana Medical School, Accra, Ghana; 3 Faculty of Trauma and Orthopaedics, Ghana College of Surgeons and Physicians, Accra, Ghana; 4 Department of Surgery, University of Medical School. Accra, Ghana; 5 Department of Statistics and Actuarial Sciences, University of Ghana, Legon, Ghana

**Keywords:** Osteoarthritis, Betamethasone, Methylprednisolone, WOMAC, VAS

## Abstract

**Background:**

Knee osteoarthritis is a chronic degenerative condition often viewed as an inevitable aspect of aging. It leads to the progressive deterioration of articular cartilage, resulting in pain and significant limitations in global movement. Intra-articular steroid injections are effective for pain control and functional improvement. This study focuses on Betamethasone Dipropionate and Methylprednisolone Acetate, both of which have shown promising outcomes, evaluated at the Korle Bu Teaching Hospital.

**Objective:**

The study aimed to compare the effectiveness of betamethasone dipropionate and methylprednisolone acetate in managing Kellgren Lawrence (KL) stage 2 to 4 primary knee osteoarthritis.

**Design:**

This was a single-blinded comparative study conducted at the Orthopaedic Clinic of a tertiary hospital

**Participants:**

Patients with osteoarthritis in one or both knees, presenting with a pain score of ≥4 on a 0–10 Visual Analogue Scale (VAS), were included.

**Main Outcome Measure:**

Effectiveness in controlling pain and improving function.

**Results:**

The average age of participants was 60 years. At two weeks, the P-values for VAS and the Western Ontario and McMaster Universities Osteoarthritis Index (WOMAC) scores between groups A and B were 0.495 and 0.927, respectively. At four weeks, these values were 0.810 and 0.372. However, by twelve weeks, the P-values for VAS and WOMAC scores were 0.026 and 0.0235, indicating significant differences.

**Conclusions:**

Both steroid injections provided symptom relief; however, Betamethasone Dipropionate demonstrated superior long-term effectiveness compared to Methylprednisolone Acetate, offering better sustained pain relief beyond eight weeks.

**Funding:**

None declared

## Introduction

Osteoarthritis of the knee is a very common form of arthritis affecting people worldwide, with a rising prevalence.^1^ It is a degenerative joint disease characterised by the breakdown of cartilage, fibrotic changes to the joint capsule, bony changes, and inflammation of the synovial membrane.^2^ Knee osteoarthritis is a chronic degenerative disease and is thought by many to be an inevitable consequence of growing old.^3^ It is characterised by a progressive deterioration of articular cartilage, resulting in pain and severe disability.^4^-^7^ It affects millions of people and is the most common cause of pain and physical disability in the world today, with huge social and economic costs.^8^ In Europe, over 40 million people are affected by OA, and it is estimated that 130 million Europeans will suffer from OA by 2050.^8^ It affects one-third of elderly people.^9^ Injury to the knee joint can also contribute to acute and chronic degradation of cartilage in the younger population.^8^ Currently, no treatment can reverse or halt OA, other than pain relief and corrective osteotomies until joint replacement becomes necessary, with considerable economic impact.^8^

Abnormal mechanical loading plays a vital role in the progression of cartilage degeneration after injury.^8^ This condition greatly limits daily activities such as sweeping, exercising, walking, stair climbing, and housekeeping, leading to a lack of functional independence and impairment of quality of life.

Corticosteroids are very potent anti-inflammatory agents.^1^ Intra-articular injections have been used for nearly five decades for the symptomatic treatment of osteoarthritis.^1^ Corticosteroids exert their anti-inflammatory action by interrupting the inflammatory and immune cascade at several levels including impairment of antigen opsonisation, interference with inflammatory cell adhesion and migration through vascular endothelium, interruption of cell-cell communication by alteration of release or antagonism of cytokines (interleukin-1), impairment of leukotriene and prostaglandin synthesis, inhibition of production of neutrophil superoxide, metalloprotease and metalloprotease activator (plasminogen activator), and decreased immunoglobulin synthesis.^6^

These will eventually lead to a reduction in pain. Various steroids such as betamethasone dipropionate, Triamcinolone Acetonide and Methylprednisolone can be used for intra-articular injections. When these agents are mixed with a local anaesthetic agent, they potentiate immediate pain relief for the patient.

Symptomatic osteoarthritis of the knee occurs in 10% of men and 13% of women aged 60 years or older.^10^ Globally, it affects about 250 million people, with over 27 million in the United States of America.^2^ African American population has a higher risk of developing knee Osteoarthritis as well as obese elderly female patients.^2^

The burden of knee osteoarthritis in developing countries tends to be higher due to late diagnosis and a lack of logistics and access to specific interventions, such as arthroplasty and rehabilitation.A population-based study in South Africa estimated the prevalence of osteoarthritis of the knee in a rural setting to be 33.1% among adults aged 35 years and older.^11^ A study in Burkina Faso reported a prevalence rate of 0.5% among Human Immunodeficiency Virus (HIV) infected adult patients on Anti-retroviral agents.^11^ Another study in Tunisia has reported a prevalence rate of 4.7% among the elderly in primary care. In developing countries such as Ghana, Nigeria, Ivory Coast, Togo, and Cameroon, chronic degenerative joint diseases are yet to be prioritised in medical services, as epidemics and tropical diseases tend to receive the highest attention in terms of logistics and resources.^12^,^13^ The prevalence of osteoarthritis in Ghana in those aged 50-59 years is 11.5%, and in those aged 60-69 years is 15.4%.^14^

There is a direct association between osteoarthritis (OA) and heavy physical occupational activity^7^. Usually, apart from joint replacement, there is no cure and management is aimed at pain relief and improvement of physical function.^15^ It is characterised by chronic pain, stiffness, and swelling that leads to reduced mobility and impaired quality of life (QoL).^16^

Osteoarthritis is usually classified as primary or secondary. Primary osteoarthritis can either be localised or generalised, and the latter is more common in post-menopausal women. Secondary osteoarthritis usually has an underlying cause such as obesity, trauma, Paget's disease, and inflammatory arthritis.^17^

In this study, the effectiveness of betamethasone dipropionate and methylprednisolone Acetate was evaluated using standardised tools, such as the VAS and the WOMAC.

## Methods

### Study design and setting

This design was a single-blinded comparative study involving patients with osteoarthritis of the knee presenting at the Orthopaedic Unit of the Department of Surgery, Korle-Bu Teaching Hospital. The study commenced on August 1, 2021, and concluded on December 30, 2021.

### Sample Size

The sample size was calculated using the formula^18^

*λ* =non-centrality parameter for repeated measurements = 10.2
f = medium effect size among the means of the pain levels over the period = 0.25
*ρ* = medium effect size for Pearson correlation coefficient = 0.3
M = number of repetitions of primary measurement outcome (i.e., pain) = 5

$$n=\frac{10.2(1-0.3)}{5\times 0.25^{2}}$$

The minimum sample size required was 23 per group. Accounting for a 10% attrition rate, the sample consisted of 25 participants per group. Hence, the total minimum sample size for this study is 50.

### Study Participants

#### a. Inclusion criteria

1. Study participants were patients aged between 45yrs and 75yrs who failed to respond to conservative treatment options of pain
2. Patients with radiological diagnosis of OA, Kellgren–Lawrence grade 2–4.
3. Patients with pain in the knee with a pain score between 9 and 20 on the WOMAC-A (Pain) scale and function between 0 and 68 on the WOMAC-C (function).
4. Patients with pain in the knee with a pain score ≥ four on VAS.

#### b. Exclusion criteria

1. Patients with a history of secondary osteoarthritis
2. Patients with a history of bleeding disorders
3. Patients with a history of comorbidities, such as diabetes, rheumatoid arthritis
4. Patients with a known history of hypersensitivity to steroids
5. Patients with severe hip pain
6. Patients with radicular spine disease

### Ethical considerations

Ethical Approval was obtained from the Institutional Review Board of the Korle Bu Teaching Hospital (Ref: KBTH-IRB/00086/2020).

### Data collection

All patients presenting at the Orthopaedic Clinic of the Korle Bu Teaching Hospital with a history and physical examination suggestive of an acute flare of osteoarthritis of the knee or severe Osteoarthritis of the knee awaiting total knee arthroplasty were selected. Standing X-rays of the affected knee in both anteroposterior and lateral views were requested. Those with X-ray features of stage 2 - 4 Kellgren and Lawrence classification together with a pain score of more than 4 on VAS and more than 9 on WOMAC were recruited into the study.

Participants were educated on osteoarthritis and the treatment modalities available. Written consent was obtained from each patient by the principal investigator. Following education on the study and available treatments. Seventy patients provided written informed consent. Twenty did not meet the inclusion criteria, resulting in a final sample of fifty participants.

Fifty unique codes were randomly generated using a computer for the participants, with each code assigned either an “A” or a “B” designation. There were twenty-five codes labelled A and twenty-five labelled B. Each code was placed in a sealed envelope. Participants were then allowed to randomly select one envelope, which they handed to the nurse. The nurse prepared the injection solution based on the selected code, assigning each patient to either Group A or Group B accordingly. Group A received betamethasone dipropionate/betamethasone sodium phosphate 2mg + 5mg/ml in 2 mL (Diprofos, Schering-Plough), and Group B received methylprednisolone acetate (Depo-Medrol, 40 mg/mL, Pfizer). Both drugs were mixed with 2 mLs of plain Marcaine and 2 mLs of 2% plain Lidocaine. The lead investigator was not blinded to the drug being used for each patient. The patients, however, were blinded. Pain severity was measured using the VAS and the WOMAC scale at baseline, 2 weeks, 4 weeks, 8 weeks, and 12 weeks after intra-articular injection.

### Procedure

In both groups, the joint was entered with an 18-gauge needle via the medial or lateral soft spot in the anterior knee and the synovial fluid if present was aspirated as much as possible. The aspirated fluid was measured and discarded, after which betamethasone or methylprednisolone was injected into the affected knee. Either betamethasone or methylprednisolone preparations were mixed with 2 mLs of plain 2% Lidocaine, a local anaesthetic agent and 2 mLs of plain Marcaine. The Lidocaine reduced any discomfort or pain due to the injections.

A nurse was assigned to set up the theatre and to prepare both drugs. All procedures were carried out in the Orthopaedic theatre at the Department of Trauma and Orthopaedics. Participants were placed in the supine position with the hip extended, and a thin pad was placed under the knee to relax the quadriceps. The skin of the injection site was cleaned with a povidone-iodine solution. The knee joint was approached laterally. Either 2mls of diprofos mixed with 2mls of 2% Lidocaine and 2mls of plain Marcaine or 1ml of 40 mg depo-Medrol mixed with 2mls 2% Lidocaine and 2mls of plain Marcaine was injected under sterile conditions using an 18G needle and 10cc syringe. Hypodermic needles were changed between drawing up the steroid and injection. The size of the syringe was uniform throughout the study. Additional injections were not permitted during the study period. The principal investigator did all injections.

All patients were given oral Paracetamol 1g three times a day for 48 hours. This mitigated the potential pain from acute steroid injection. Patients were assessed at baseline, four weeks, eight weeks, and twelve weeks after injection. The two main variables, pain and function, were measured using the VAS and the WOMAC. To avoid inter-observer variability, patients were assessed pre- and post-intervention by the principal investigator. Complications from the intra-articular knee injection, such as infection, hot flashes, skin pigmentation, and subcutaneous fat atrophy, were monitored. The entire procedure adhered to strict aseptic protocols. Throughout the study and during follow-up visits, no instances of septic arthritis were reported.

Patients were contacted via telephone calls and WhatsApp messages. Each participant was reviewed and examined 4 times during the study period. The primary outcome measured was a reduction in pain at the affected knee joint and an improvement in function, as assessed using VAS and WOMAC, respectively.

### Data Analysis

The data was analysed in SPSS version 25. Categorical variables were summarised by their number and relative frequencies. Continuous variables following a normal distribution were summarised by mean and standard deviation. Alternatively, non-normally distributed variables were summarised with their median, interquartile range, minimum, and maximum. Comparisons in continuous variables between treatment groups were made using Student's t-test or the Mann–Whitney U test for normally or non-normally distributed variables, respectively.

Analysis of pain levels between the two groups (A and B) was done using One-Way Analysis of Variance (ANOVA). Adequacy of pain reduction derived from using VAS and WOMAC was compared using the Chisquare test.

Continuous variables, such as age and pain, were summarised as means (standard deviations). Categorical variables such as gender, occupation and complications were summarised as proportions, tables, and graphs. P-value ≤ 0.05 was considered statistically significant.

## Results

### Demographics of Patients

In all, 50 patients were studied. The mean age of the cohort was 60 years. In Groups A and B, the mean ages were 61.72 ± 10.10 and 58.76 ± 7.15 years, respectively (Table 1). According to the study, 16% of the participants are males and 84% are females. Sixty-six per cent (66%) of the patients were obese, 20% were overweight, and 14% had a normal weight. The two groups were similar in terms of age, weight and BMI as shown in Table 1.

**Table 1:** Demographics statistics of Group A and Group B

	Mean ±Standard deviation		P-values
	A	B	
Age	61.72±10.10	58.76±7.15	0.0725
Weight	83.42±16.45	86.90±15.77	0.322
BMI	31.82±8.01	33.45±6.50	0.2165

**Figure 2:**
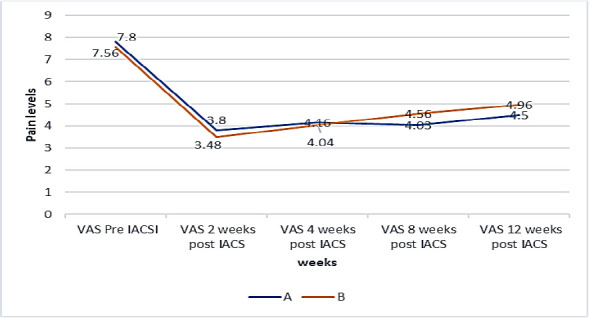
Trends of reduction of the level of pain for the VAS of Group A and Group B

Over the study period, the mean pain score on the VAS for patients in group A decreased from a baseline of 7.8 to 3.8 in the second-week assessment of the study. The score, however, increased to 4.16 in week four and to 4.5 in week 12. For group B, the VAS mean score decreased from 7.56 in the pre-study period to 3.48 in week two. The score increased in group B gradually from 4.04 in week four to 4.96 in week 12. (Figure 2):

The pain levels were similar in both groups from baseline (VASPreIACSI) through week 2 to week 8 (PostIACSI), as shown by the mean VAS in Table 2 (P > 0.05). However, pain levels were significantly different in the two groups at week twelve post IACSI (p=0.026)

**Table 2:** Mean VAS and WOMAC Between the two Groups at Each Stage Over the Period of the study

VAS	Total	Mean VAS Pre IACSI	Mean VAS 2 weeks post IACS	Mean VAS 4 weeks post IACS	Mean VAS 8 weeks post IACS	Mean VAS 12 weeks post IACS
A	25	7.8	3.8	4.16	4.03	4.5
B	25	7.56	3.48	4.04	4.56	4.96
P-value		**0.252**	**0.469**	**0.81**	**0.062**	**0.026**
**WOMAC**	**Total**	**Mean WOMAC Pre- IACS**	**Mean WOMAC 2 weeks post IACS**	**Mean WOMAC 4 weeks post IACS**	**Mean WOMAC 8 weeks post IACS**	**Mean WOMAC 12 weeks post IACS**
A	25	16.67	7.52	7.08	7.24	7.48
B	25	15.48	7.44	7.88	7.68	8.8
P-value		**0.689**	**0.927**	**0.372**	**0.192**	**0.0235**

• VAS(visual analogue scale, WOMAC A (McMaster Universities Osteoarthritis Index) IACSI (Intra articular corticostroid injection)

Table 2 shows that the baseline pain for participants using VAS was similar in the two groups(p=0.252) and the function in both groups was also similar (p=0.689)

### Trends of mean pain score on WOMAC-A-A for the participants in Groups A and B

**Figure 3:**
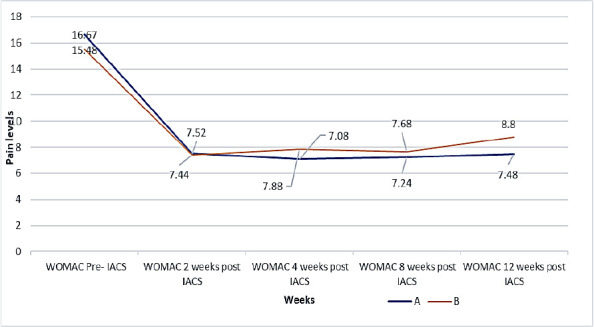
Trends of reduction of the level of pain for the WOMAC of Group A and Group B

The mean pain score on WOMAC for patients in group A decreased from 16.72 to 7.52 in the second week of the study. The score decreased to 7.08 and 7.24 in the fourth and eighth weeks, respectively, and then increased to 7.48 in the twelfth week. For group B, the WOMAC mean score decreased from 15.48 in the pre-study period to 7.44 in week two. The score gradually increased to 7.88 in week four to 8.88 in week twelve. The trend levels were similar. In both groups, using the WOMAC scale, other than week twelve, where the pain score was lower in Group A than in Group B.(Figure 3)

### The Knee Function of Patients Using WOMAC-C

According to the study, both groups showed improvement in function; however, patients in Group A experienced a superior improvement in function. The difference in improvement of function in both groups was not statistically significant between the two groups for the eight weeks but became statistically significant in the twelfth week (P-value=0.041). Lower scores meant improved function.

There was a gradual decrease in WOMAC-C scores for both groups from the beginning of the study to the twelfth week. Lower WOMAC-C scores meant improvement of function. Patients in group A showed better functional improvement throughout the study compared to those in group B. The pre-injection pain scores were severe in both groups, with a VAS score (7-10) in 88% of patients and a moderate VAS score (4-6) in 12% of patients. Similarly, 15 patients had pain levels on the WOMAC scale within 11-15, and 10 patients had pain levels on the WOMAC scale within 16-20 (WOMAC).

At two weeks, the mean pain score of 7.8 prior to the study, as measured on the VAS scale, had decreased to 3.8 for group A, representing a 48.7% reduction in pain, and from 7.56 to 3.48 for group B, representing a 46.0% reduction in pain. On the WOMAC scale the mean pain score decreased from 16.72 to 7.52 for group A representing a 45.0% reduction in pain and for group B from 15.48 to 7.44 representing 48.1%.

At four weeks, the mean pain score on VAS increased from 3.8 to 4.16 for group A, representing a 9.5% increment in pain, and from 3.48 to 4.04 for group B, representing a 16.1% increment in pain. This increment in pain was still less than the pain level before the study. Additionally, on the WOMAC, the mean pain score had decreased from 7.52 at two weeks to 7.08 at four weeks for group A, representing 5.6%, and for group B, pain increased from 7.44 to 7.88, representing 5.9%. At eight weeks, the mean pain score on VAS for group A decreased from 4.16 to 4.03, representing a 3.1% decrease in pain level. For Group B, the level of pain increased from 4.04 to 4.56, representing a 12.9% increment in the level of pain.

The mean pain score on the WOMAC level of pain increased from 7.08 in the fourth week to 7.24 in group A, representing a 2.3% increase, and decreased from 7.88 in the fourth week to 7.68 in group B, representing a 2.5% decrease. At twelve weeks, the mean pain score on the VAS scale increased from 4.03 to 4.5, representing an 11.7% increment in pain for group A and from 4.56 to 4.96, representing an 8.9% increment for group B. The P value was 0.026, which was less than 0.05. On the WOMAC- A mean pain score, pain increased from 7.24 to 7.48, representing 3.3% for group A and from 7.68 to 8.8, representing 14.6% for group B. The P value of 0.0235 was less than 0.05. From the test for equality of means, the P values for VAS and WOMAC were 0.016 and 0.019, respectively, for both groups. Both P-values were less than 0.05. Using the WOMAC-A scale, the percentage reduction in pain from baseline to week twelfth for Group A was 55.3%, and that of Group B was 43.2.7%.

The pre-injection pain scores were severe in both groups, with a VAS score (7-10) in 88% of patients and a moderate VAS score (4-6) in 12% of patients. Similarly, 15 patients had pain levels on the WOMAC scale within 11-15, and 10 patients had pain levels on the WOMAC scale within 16-20 (WOMAC).

At two weeks, the mean pain score of 7.8 before the study on the VAS scale had decreased to 3.8 for group A, representing a 48.7% reduction in pain, and from 7.56 to 3.48 for group B, representing a 46.0% reduction in pain. On the WOMAC scale, the mean pain score decreased from 16.72 to 7.52 for group A, representing a 45.0% reduction in pain, and from 15.48 to 7.44 for group B, representing a 52.1% reduction.

At four weeks, the mean pain score on VAS increased from 3.8 to 4.16 for group A, representing a 9.5% increment in pain, and from 3.48 to 4.04 for group B, representing a 16.1% increment in pain. This increment in pain was still less than the pain level before the study. Additionally, on the WOMAC, the mean pain score had decreased from 7.52 at two weeks to 7.08 at four weeks for group A, representing 5.6%, and for group B, pain increased from 7.44 to 7.88, representing 5.9%.

At eight weeks, the mean pain score on VAS for group A decreased from 4.16 to 4.03, representing a 3.1% decrease in pain level. For Group B, the level of pain increased from 4.04 to 4.56, representing a 12.9% increment in the level of pain.

The mean pain score on the WOMAC level of pain increased from 7.08 in the fourth week to 7.24 in group A, representing a 2.3% increase, and decreased from 7.88 in the fourth week to 7.68 in group B, representing a 2.5% decrease. At twelve weeks, the mean pain score on the VAS scale increased from 4.03 to 4.5, representing an 11.7% increment in pain for group A and from 4.56 to 4.96, representing an 8.9%. The P-value was 0.026, which was less than 0.05.

On the WOMAC- A mean pain score, pain increased from 7.24 to 7.48, representing 3.3% for group A and from 7.68 to 8.8, representing 14.6% for group B. The P value of 0.0235 was less than 0.05. From the test for equality of means, the P values for VAS and WOMAC were 0.016 and 0.019, respectively, for both groups. Both P-values were less than 0.05. Using the WOMAC-A scale, the percentage reduction in pain from baseline to week twelfth for Group A was 55.3%, and that of Group B was 43.2.7%.

## Discussion

From this study, the majority (84%) of the study population with OA were females, with a male-to-female ratio of 1:5. This finding is similar to that reported by Buyuk AF et al.^17^ in their study. In that study, the male-to-female ratio was 4:1. Davalillo et al., in a similar study, compared the efficacy of Betamethasone Dipropionate and Hyaluronic acid, reporting a male-to-female ratio of 3:2^19^. Zyan Y et al. and Hunter D et al. also noted the high prevalence of osteoarthritis in females.^10^,^20^ In this study, the mean age of the study population was 60 years. This confirms the fact that primary OA is common in the elderly population. This is like the finding of Umut et al 21, who also did a similar study. They reported that the mean age was 60 years. Davalillo et al. also reported a mean age of 60 years, with a male-to-female ratio of 1:2. Haider Mohammad Ziaul et al.^22^, in their study titled “Risk factors of knee osteoarthritis in Bangladeshi adults: a national survey,” reported a mean age of patients with osteoarthritis as 51.7 years. They studied a total of 1,843 people, comprising 892 males and 951 females. This finding differed from the study's findings. This may be due to our small sample size compared to theirs. Prieto et al. 23 also noted that the disease is common in individuals aged 67 years and older. From the study, 66% (33) of the patients were obese, 20% (10) were overweight, and 14% had a normal weight. This is consistent with the Chingford Study^24^, which confirmed that excess body weight is a powerful predictor of OA of the knee. It is also like what Zheng et al reported, that being overweight or obese was approximately 2.5 and 4.6 times more likely to have knee OA than having a normal weight.^25^ From the study, the total number of knees injected with either betamethasone dipropionate or methylprednisolone was sixteen more than the total number of fifty patients recruited into the study. This was because sixteen of the patients recruited had bilateral disease. Radiologically, the grades of osteoarthritis using the Kellgren–Lawrence Scale were similar, and there was no statistically significant difference found between the two groups. This finding is identical to what Umut et al found in their study.^21^ Davalillo et al. reported a similar finding in their study. In their study, there were no differences between groups in terms of KL grade or the affected knee.

Patients who were treated with betamethasone dipropionate had a significant reduction in pain compared to those who were treated with methylprednisolone. At two weeks, the Levene's test of equality, which compared the effectiveness of the two drugs in terms of pain reduction using the two tools, yielded P values of 0.629 (VAS) and 0.453 (WOMAC). These values were greater than 0.05. This indicated that there was no difference in variation between the two groups in terms of pain reduction.

At four weeks, the Levene's test of equality's P-value was 0.277 for VAS and 0.18 for WOMAC, respectively. The values were greater than 0.05. This indicated that there was no statistical difference between the two drugs concerning the reduction in pain. At eight weeks, the Levene's test for the VAS was 0.123, and WOMAC was 0.155. These values were greater than 0.05, which meant that there was no statistically significant difference between the two drugs. The P-value for the VAS between the two groups was 0.062, which is less than 0.05. This meant that at eight weeks, Betamethasone dipropionate offered better pain relief than methylprednisolone on the VAS score.

At twelve weeks, even though the level of mean pain on both the VAS and WOMAC scales increased steadily from the second week to the twelfth week, the level of pain did not get to the pre-injection level. This finding is also similar to that of the study of Umut et al.^21^ In that study, the pain level on the VAS decreased significantly in the first 3 weeks, from 7.7 to 4.3, and then increased gradually to 5.5 in the twelfth week. By the end of that study the pain level at 12 weeks never reached the pre injection pain. They also reported a 26% reduction in the percentage of betamethasone dipropionate and a 35.1% reduction in the percentage of methylprednisolone. Using the VAS score, the percentage reduction in pain for Group A from the baseline to the twelfth week was 42.3% and for Group B, 34.4%. This report is similar to the Umut et al study.^21^ The difference in outcome may be due to the brand of drugs used and patient characteristics in terms of grade of osteoarthritis using the Kellgren and Lawrence classification.

In this study, it was thus observed that patients in both groups benefited from an intra-articular injection. Both steroid types had similar effects and durations of efficacy in the first eight weeks; however, betamethasone dipropionate provided a longer duration of pain relief, both clinically and statistically. This finding differs from what Umut et al. reported.^21^ They reported that for early symptomatic relief, methylprednisolone is the first choice of drug.

There is scanty data comparing the efficacy of the two drugs used as a depo for managing pain in primary osteoarthritis of the knee. Buyuk, Abdul Fettah et al^17^ in 2017 compared methylprednisolone and triamcinolone acetate and concluded that both drugs are effective, and they peak in the first two weeks. This is comparable to what this study revealed. From the study, the maximum effect of the drug was seen in the first two weeks. This is similar to what Abdul et al in their study. According to the study by Umut et al., the maximum effect of the drugs occurred within the first 1 to 3 weeks.^21^ Kewei Tian et al.^26^ in their 2018 study, titled “Intra-articular injection of methylprednisolone for reducing pain in knee osteoarthritis,” concluded that Intra-articular methylprednisolone injection was associated with improved pain relief.

For Physical Function, the study showed that both drugs offered patients improvement in the physical function of the knee joint. This finding is consistent with what Umut et al^21^ reported in their study titled “Efficacy comparisons of the intraarticular steroidal agents in the patients with knee osteoarthritis”. They noted that intra-articular injections of both Betamethasone and methylprednisolone improve function. Davalillo et al. also reported that intra-articular betamethasone dipropionate improves function.^19^ This improvement in physical function (17 items, scored 0-68) was evidenced by the decrease in WOMAC scores from baseline to the twelfth week. At two weeks, there was a 26.4% and 21.3% improvement in function for Groups A and B, respectively. The improvement in function of both groups increased throughout the study period. In Group A, the function improved to 37.9% at four weeks, 48.3% at eight weeks and 51.5% at twelfth weeks. For Group B, the function improved to 31.0% at four weeks, 44.5% at eight weeks and 47.8% at twelfth weeks. Patients in group A had better physical function throughout the study as compared to those in group B. The difference in function at two weeks and four weeks was not statistically significant, as the P-value was greater than 0.05. However, at eight weeks and twelve weeks, the difference in improved function was statistically significant between the groups, as evidenced by P values of less than 0.05. Patients in group A had better function compared to those in Group B.

In a similar study by Umut et al, both drugs had comparable effectiveness in terms of function at three months.^21^ In that study, 30 patients received Betamethasone Dipropionate, and 30 patients received Methylprednisolone. This finding differed from what the study found at the twelfth week. This may be due to the strength of Betamethasone used in that study. A dosage of 3mg, 1ml was used in that study. Davalillo, et al. in their study, reported a 48.5% improvement in physical function in patients who received betamethasone dipropionate.^18^ This is similar to our study, which found a 51.5% improvement in function in those who received betamethasone dipropionate.

The study was conducted over 12 weeks, and the exact duration of efficacy of the drugs could not be determined within that timeframe. In the study by Buyuk et al., the effect of methylprednisolone persisted for 24 weeks after the injection. This study can be conducted in multiple hospitals on a larger scale over an extended period.^17^ The findings from that study can help surgeons in West Africa consider betamethasone dipropionate as a first-choice drug in intra-articular injections in managing pain in primary osteoarthritis of the knee. During the study period, no side effects from the steroid injection, such as infection, skin hypopigmentation, or skin atrophy, were recorded.

This study had some limitations, and it is important to mention them. This study did not compare the efficacy of the two drugs beyond twelve weeks and could not determine at which time patients can receive a second injection in relapse. In a similar study by Wang et al.^27^ they reported that there was no significant difference in the VAS score at month 6 compared with the baseline in either group.

## Conclusion

A single dose of methylprednisolone acetate or betamethasone dipropionate provided symptomatic pain relief and improved functionality in patients with primary osteoarthritis of the knee. Betamethasone dipropionate demonstrated a better long-term effectiveness than methylprednisolone acetate, with sustained clinical efficiency levels in a significant number of patients twelve weeks after administration. Both steroid types had similar effects and duration of efficacy in the first eight weeks, but betamethasone dipropionate provided a longer duration of pain relief both clinically and statistically

## Figures and Tables

**Figure 1:**
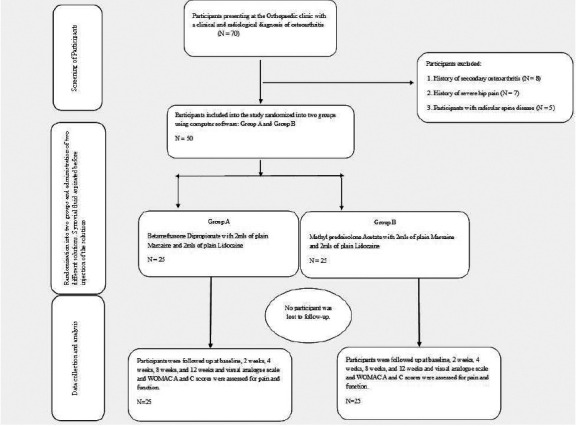
Flowchart showing data collection and procedure

**Figure 4:**
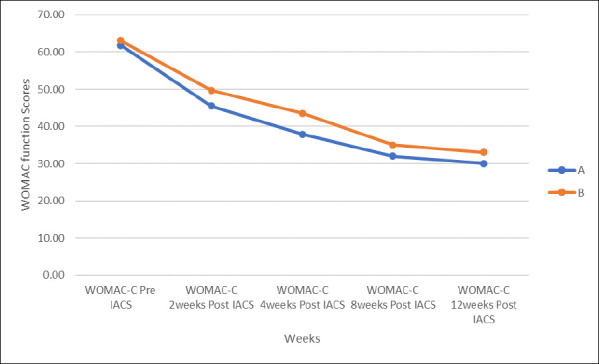
Knee function of patients using the WOMAC-C Scale for Groups A and B
